# Supplementary material for: The kinetic energy of PAH dication and trication dissociation determined by recoil-frame covariance map imaging
Source: Phys Chem Chem Phys. 2022 Jun 20;24(38):23096–105. doi: 10.1039/d2cp02252d (PMC9533308; doi:10.1039/d2cp02252d)
Supplement: CP-024-D2CP02252D-s001 [file CP-024-D2CP02252D-s001.pdf]

Electronic Supplementary Material (ESI) for Physical Chemistry Chemical Physics.
This journal is © the Owner Societies 2022

Supporting information for:

The Kinetic Energy of PAH Dication and Trication Dissociation Determined by Recoil-Frame Covariance Map Imaging

J. W. L. Lee,[a,b] D. S. Tikhonov,[a,c] F. Allum,[b] R. Boll,[d] P. Chopra,[a,c] B. Erk,[a] S. Gruet,[a] L. He,[a] D. Heathcote,[b] M. M. Kazemi,[a] J. Lahl,[e] A. K. Lemmens,[f,g] D. Loru,[a] S. Maclot,[h,i] R. Mason,[b] E. Müller,[a] T. Mullins,[j] C. Passow,[a] J. Peschel,[e] D. Ramm,[a] A. L. Steber,[a,c,k] S. Bari,[a] M. Brouard,[b] M. Burt,[b] J. Küpper,[l,k,j] P. Eng-Johnsson,[e] A. M. Rijs,[f] D. Rolles,[m] C. Vallance,[b] B. Manschwetus,[a] M. Schnell[a]

1 Computational procedure

1.1 General computational details

Computation of the dissociation mechanisms of the dication and trication of the fluorene was performed using an augmented Born-Oppenheimer molecular dynamics (aBOMD) approach,[1–4] where the motion of the nuclei is governed by the ground electronic state gradients, and the electronic excitation is represented *via* an internal excess energy (IEE), external energy reservoir. The IEE energy is assigned to the molecule using a recently proposed semi-heuristic model.[4] Following IEE assignment, the energy is redistributed into the vibrational degrees of freedom with a rate given by the internal conversion (IC) constant, which was computed using a classical electron-nuclei collision model.[4] The potential energy surfaces were computed through the GFN2-xTB method[5] from the XTB semi-empirical package,[6] which has been shown to work well for molecular fragmentation dynamics.[3, 4] A detailed description of the computational methods used can be found in the PyRAMD manual,[7] and the source code is also available for download.[8]

Fragmentation dynamics were computed for fluorene with a time step of 1 fs covering a total time of 10 ps in each BOMD trajectory. The XUV or XUV and IR pulses were applied simultaneously at the first step of the simulation.

1.2 Details of the aBOMD approach

1.2.1 Initial conditions sampling

In PyRAMD, we use an intermediate initial conditions sampling variant: beyond standard classical Maxwell-Boltzmann sampling, but simpler than standard Wigner sampling from the vibrational wavefunctions in the harmonic oscillator approximation. We start from the assumption that for each degree of freedom of each nucleus at temperature $T = 0$ K, we need to fulfill the Heisenberg inequality for the position and momentum:

$$\Delta x_0 \cdot \Delta p_0 \geq \frac{\hbar}{2} \ .$$

[a] *Deutsches Elektronen-Synchrotron DESY, Germany*
[b] *Department of Chemistry, University of Oxford, United Kingdom*
[c] *Institute of Physical Chemistry, Christian-Albrechts-Universität zu Kiel, Germany*
[d] *European XFEL, Germany*
[e] *Department of Physics, Lund University, Sweden*
[f] *Radboud University, FELIX Laboratory, The Netherlands*
[g] *Van't Hoff Institute for Molecular Sciences, University of Amsterdam*
[h] *KTH Royal Institute of Technology, Sweden*
[i] *Physics Department, University of Gothenburg, Sweden*
[j] *Department of Physics, Universität Hamburg, Germany*
[k] *Center for Ultrafast Imaging, Universität Hamburg, Germany*
[l] *Center for Free-Electron Laser Science CFEL, Deutsches Elektronen-Synchrotron DESY, Germany*
[m] *J.R. Macdonald Laboratory, Department of Physics, Kansas State University, KS, USA*

where Δx_0 and Δp_0 are the uncertainties for the position and momentum of the particle at $T = 0$ K. This can be achieved by sampling each coordinate (x) and momentum (p) from two Gaussian distributions:

$$\begin{cases} x \sim |\psi(x)|^2 \propto \exp\left(-\frac{(x-x_0)^2}{2\Delta x_0^2}\right), \\ p \sim |\phi(p)|^2 \propto \exp\left(-\frac{p^2}{2\Delta p_0^2}\right), \end{cases}$$

where x_0 is the initial coordinate value at a given XYZ structure, and we assume that initially each particle is motionless. This is equivalent to sampling the coordinates and momentums from the Wigner function:

$$W(x,p) = \frac{1}{\pi\hbar}\int_{-\infty}^{+\infty} \psi(x+\xi)\exp\left(2i\frac{p\xi}{\hbar}\right)\psi(x-\xi)d\xi$$

for the Gaussian wavepacket localized around x_0:

$$\psi(x) \propto \exp\left(-\frac{(x-x_0)^2}{4\Delta x_0^2}\right) ,$$

that is:

$$W(x,p) \propto \exp\left(-\frac{(x-x_0)^2}{2\Delta x_0^2} - \frac{2\Delta x_0^2 p^2}{\hbar^2}\right) .$$

To fulfill the Heisenberg inequality, we take the uncertainties parameterized as the following:

$$\Delta x_0 = \sqrt{\alpha \cdot \frac{\hbar\tau}{2m}}$$

and

$$\Delta p_0 = \sqrt{\frac{\hbar m}{2\tau}}$$

where m is the mass of the nuclei, τ is the arbitrarily chosen time, and $\alpha \geq 1$ is an arbitrarily chosen scaling coefficient. If $\alpha = 1$ the inequality turns into equality. These parameterizations correspond to the minimal spreading Gaussian wave packet for the time τ. For a hydrogen atom ($m = 1$ a.m.u.) at $\tau = 1$ fs and $\alpha = 1$, this yields $\Delta x_0 = 0.06$ Å.

We approximate the effect of temperature on the momentum of the particle by adding extra momentum p' ($p \to p + p'$) distributed according to a Maxwell-Boltzmann distribution ($p' \sim \exp\left(-\frac{\beta p'^2}{2m}\right)$, where $\beta^{-1} = k_{\mathrm{B}}T$). This leads to a momentum distribution:

$$f(p) \propto \int_{-\infty}^{+\infty} \exp\left(-\frac{\beta p'^2}{2m}\right)|\phi(p+p')|^2 dp' = \int_{-\infty}^{+\infty} \exp\left(-\frac{\beta p'^2}{2m} - \frac{(p+p')^2}{2\Delta p_0^2}\right) dp' .$$

After integration over p', we get the distribution:

$$f(p) \propto \exp\left(-\frac{p^2}{2\Delta p_T^2}\right) ,$$

where

$$\Delta p_T^2 = \Delta p_0^2 + m k_{\mathrm{B}} T ,$$

i.e. instead of pure Maxwell-Boltzmann sampling, we have a sampling with adjusted temperature $T_{\mathrm{eff}} = \Delta p_0^2/(m k_{\mathrm{B}}) + T$. By setting $\Delta x_0 = 0$ and $\Delta p_0 = 0$, we obtain the classical Maxwell-Boltzmann sampling routine.

In our the simulations, we applied the default PyRAMD values of $\tau = 10$ fs, $\alpha = 1$, and $T = 0$ K.

1.2.2 Ionization and excitation procedure

Upon interaction with the laser pulses, we assume that the molecule M absorbs $N_{\mathrm{ph}} \geq 0$ photons γ, emits $N_{\mathrm{els}} \geq 0$ electrons (e^-), resulting in an excited ionic state $\left(\mathrm{M}^{N_{\mathrm{els}}+}\right)^*$. Such interaction can be described *via* a pseudo-chemical reaction:

$$\mathrm{M} + N_{\mathrm{ph}} \times \gamma \to \left(\mathrm{M}^{N_{\mathrm{els}}+}\right)^* + N_{\mathrm{els}} \times \mathrm{e}^- .$$

The excitation energy of the $\left(\mathrm{M}^{N_{\mathrm{els}}+}\right)^*$ with respect to the ground ionic electronic state $\mathrm{M}^{N_{\mathrm{els}}+}$ is the previously described internal excess energy (IEE).

The integer numbers N_{ph} and N_{els} are sampled on the fly from the Poisson-distribution, where the chance of getting the result $n = 0, 1, 2, \ldots$ is given as $p_n = \frac{\lambda \cdot \exp(-\lambda)}{n!}$ with parameter $\lambda = \langle n \rangle$ being the mean n. The excitation energy obtained by the molecule from the laser is thus given as $E_{\mathrm{exc}} = N_{\mathrm{ph}} \cdot h\nu$, where ν is the laser pulse frequency.

The parameters λ for the N_{els} and N_{ph} in the simulations were the following:

- For the XUV pulse: $\lambda_{\mathrm{ph}} = \langle N_{\mathrm{ph}} \rangle = 1$, $\lambda_{\mathrm{els}} = \langle N_{\mathrm{els}} \rangle = 2$, $\nu = 40.9$ eV.

- For the IR pulse (when used): $\lambda_{\text{ph}} = \langle N_{\text{ph}} \rangle = 5$, $\lambda_{\text{els}} = \langle N_{\text{els}} \rangle = 0.5$, $\nu = 1.5$ eV.

The IEE assignment algorithm operates as follows:

- Two main parameters are generated from the Poisson distribution: excitation energy provided by the laser pulse $E_{\text{exc}} = N_{\text{ph}} \cdot h\nu$, and N_{els}. The parameter `IPStretching` is provided by the user. This parameter gives a threshold for the molecular excitation, by how much it can exceed the IP without emitting the electrons (i.e. if $E_{\text{exc}} \leq \text{IP} \cdot (1 + \text{IPStretching})$ with IP being the ionization potential, and $N_{\text{els}} = 0$, the molecule stays in the neutral state). We used `IPStretching`=0.2.

- The IPs and HOMO-LUMO gaps of the molecule are calculated to find the maximal ionization that can be reached, applying the restrictions that the number of removed electrons n cannot exceed N_{els}, and the required energy for ionization of n electrons cannot exceed E_{exc}. If $N_{\text{els}} = 0$ but the E_{exc} exceeds the threshold $\text{IP} \cdot (1 + \text{IPStretching})$, then one electron will be removed.

- In the final ionic state, the IEE is assigned by the following rules:
  - If no electrons were removed ($n = 0$), the excitation will increase the IEE to the value by E_{exc}. However, if E_{exc} is smaller than the HOMO-LUMO gap of the molecule, the additional IEE = 0.
  - If the ionization occurs, the IEE will be drawn from the beta-distribution, such as the probability density for getting the $\text{IEE} = \text{IEE}_{\max} \cdot x$ ($x \in [0; 1]$) given by:

$$f(x) = \frac{x^{\frac{D}{2}-1} \cdot (1-x)^{\frac{3N_{\text{els}}}{2}-1}}{B\left(\frac{3N_{\text{els}}}{2}, \frac{D}{2}\right)}$$

    where B is the beta-function, D is the scaling law for the density of states (taken as $D = 3$), and $\text{IEE}_{\max} = E_{\text{exc}} - \text{EI}$, where EI (energy of ionization) is the sum of IPs to remove n electrons.[4]

1.2.3 On-the-fly internal conversion rate estimation

The heuristic model for the internal relaxation extends a model introduced in previous work ([4]):

- electrons and nuclei are modelled as two gases,
- the electrons are modelled as hot gas and the nuclei are modelled as cold gas,
- the excess energy of electrons (IEE) is converted to the nuclei.

The rate of the relaxation, k, is given by the equation for the collision period τ (see e.g. [9])

$$k^{-1} = \tau = \frac{(m_{\text{e}} + m_{\text{n}})^2}{m_{\text{e}} m_{\text{n}}} \frac{1}{\kappa z} ,$$

where m_{e} and m_{n} are the masses of the electrons and the nucleus, κ is a dimensionless parameter, and z is given by equation:

$$z = n_{\text{e}} \cdot \sigma_{\text{en}} \cdot \sqrt{\frac{kT}{m_{\text{e}}}}$$

with n_{e} being the concentration of the electrons, σ_{en} is the collisional cross section, and T is the temperature of the electronic gas. The internal excess energy (IEE) is taken to be proportional to temperature ($\text{IEE} \propto T$).

From here, a set of heuristic assumptions are applied. First, the total rate of IC is taken as $k_{\text{IC}} = \sum_n \frac{1}{\tau_n}$, where n enumerates nuclei, and the τ_n is the relaxation time for the n-th nucleus. In each τ_n, we assume the cross sections, σ, to be proportional to the nuclear mass as $\sigma_n = \sigma_0 m_n / m_{\text{amu}}$, with σ_0 representing shared cross section per unit of mass, and m_{amu} is the atomic mass unit. The concentration of electrons we will take as $n_{\text{e}} = N_{\text{e}} / V_{\text{mol}}$ with N_{e} being the total number of elections, and V_{mol} being the molecular volume. The total volume of the molecule is approximated as:

$$V_{\text{mol}} = \sigma_0 \cdot \overbrace{\frac{1}{2} \sum_n \sum_m Z_n Z_m R_{nm} \cdot C_{nm}}^{L_{\text{mol}}} = \sigma_0 L_{\text{mol}} ,$$

where Z_n is the charge of the n-th nucleus in atomic units, R_{nm} is the distance between nuclei, and C_{nm} is the connectivity matrix of the nuclei with:

$$C_{nm} = \begin{cases} 1, & \text{if } R_{nm} \leq s \cdot (R_n + R_m), \\ 0, & \text{if } R_{nm} > s \cdot (R_n + R_m), \end{cases}$$

where R_n and R_m are the covalent radii of the atoms. Physically, this approximation represents the molecular volume as a set of cylinders located along the bonds, the lengths of the cylinder as the bond distance, and the cross section

is σ_0. By taking the ratio of masses to be $\frac{m_\mathrm{e} m_\mathrm{n}}{(m_\mathrm{e}+m_\mathrm{n})^2} \approx \frac{m_\mathrm{e}}{m_\mathrm{n}}$ (since $m_\mathrm{n} \gg m_\mathrm{e}$), we get the following expression for the IC rate:

$$k_\mathrm{IC} = \kappa \frac{\sqrt{m_\mathrm{e}} \cdot \mathrm{IEE}}{m_\mathrm{amu} \cdot L_\mathrm{mol}} N_\mathrm{e} N_\mathrm{n} \ ,$$

where N_n is the total number of nuclei in the system. To stabilize this equation if no bonds are found (i.e. all $C_{nm} = 0$), we include a stabilizing term to the denominator (L_0). This yields the final equation:

$$k_\mathrm{IC} = \kappa \frac{\sqrt{m_\mathrm{e}} \cdot \mathrm{IEE}}{m_\mathrm{amu} \cdot (L_0 + L_\mathrm{mol})} N_\mathrm{e} N_\mathrm{n} \ .$$

L_0 was varied manually, whilst κ was optimized using a training set of data from literature values [10, 11] for the lifetimes of short lived cations obtained using XUV pulses with the following reaction:

$$\mathrm{Molecule} + \mathrm{XUV} \rightarrow \mathrm{Molecule}^{+,*} + \mathrm{e}^- \ .$$

An acceptable result was found for $L_0 = 10$ [Bohr] ≈ 5[Å] and $\kappa = 1.2815616845$. The error-weighted RMSD for the training set was calculated to be 11.3 fs, and 11.4 fs for the test set. (see Figure 1). More details on the fitting

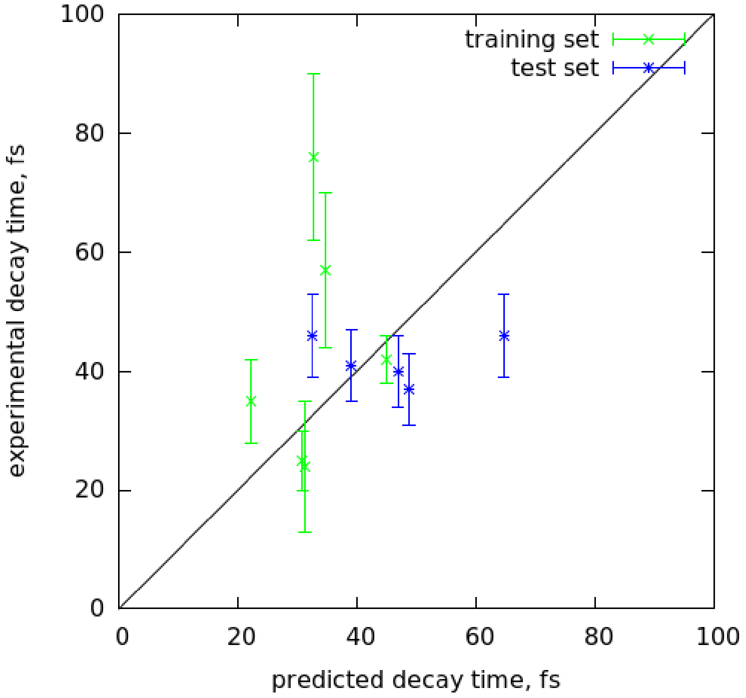

Figure 1: Comparison of the experimental and predicted internal conversion rate lifetimes for the training and test sets of the molecules.

procedure can be found in the PyRAMD repository: `https://stash.desy.de/projects/PYRAMD/repos/pyramd/browse/addData/FitDynamicalStuff/NewGasCollisionModel`.

1.2.4 Calculation of the Kinetic Energy Release

The fragmentation procedure used in PyRAMD is described in Ref. [4]. Here, we outline the procedure for calculating the kinetic energy release (KER). Let us assume that the molecule AB dissociates into fragments A and B (AB $\rightarrow$ A + B). We calculate the relative velocity of the fragments in the direction that connects their centers of mass (CoM) $\mathbf{R}_\alpha = \frac{\sum_i m_i \mathbf{r}_i}{\sum_i m_i}$ (r_i and m_i are the position and the mass of the i-th atom of fragment α =A,B). First, we find the direction connecting the CoM(A) and CoM(B), given as $\mathbf{n}_\mathrm{AB} = \frac{\mathbf{R}_\mathrm{A} - \mathbf{R}_\mathrm{B}}{|\mathbf{R}_\mathrm{A} - \mathbf{R}_\mathrm{B}|}$. Then, we project the translational velocity of each fragment ($\mathbf{V}_\alpha = \frac{\sum_i m_i \mathbf{v}_i}{\sum_i m_i}$, v_i is the velocity and the mass of the i-th atom of fragment α =A,B) on the direction connecting the CoM(A) and CoM(B) obtaining $V_\mathrm{A} = \mathbf{n}_\mathrm{AB} \cdot \mathbf{V}_\mathrm{A}$ and $V_\mathrm{B} = \mathbf{n}_\mathrm{AB} \cdot \mathbf{V}_\mathrm{B}$. The kinetic energy along the dissociation direction $\mathbf{n}_\mathrm{AB}$ is thus given by the formula KE $= \frac{1}{2}\mu(V_\mathrm{A} - V_\mathrm{B})^2$, where $\mu = \frac{M_\mathrm{A} M_\mathrm{B}}{M_\mathrm{A} + M_\mathrm{B}}$ is the reduced mass of the fragments A and B. The dissociation energy (DE) for channel AB $\rightarrow$ A + B is given by the formula DE $= E(\mathrm{A}) + E(\mathrm{B}) - E(\mathrm{AB})$, where E is the electronic energy of the particle in the parentheses. The resulting kinetic energy release (KER) along the dissociation direction is thus given as KER = KE − DE (see Figure 2), and if KER > 0, then the fragmentation is possible. Upon dissociation, the momenta of the fragments are recorded and used to compute the KER. The KER energy will be distributed between fragments A and B in accordance with the momentum and energy conservation laws. The momentum conservation gives us $p_\mathrm{A} + p_\mathrm{B} = 0$, where "$p$" is the

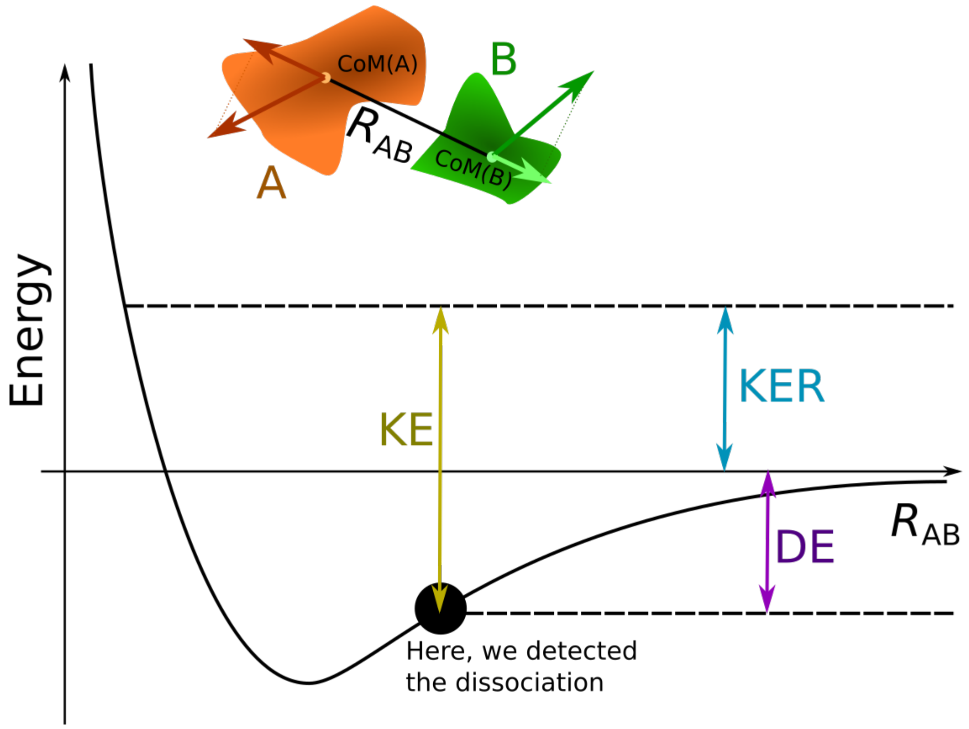

Figure 2: Schematic of the kinetic energy release (KER) calculation procedure. More details are described in the text.

momentum of the fragment along the $\mathbf{n}_{\mathrm{AB}}$ axis. Kinetic energy conservation $\frac{p_{\mathrm{A}}^2}{2M_{\mathrm{A}}} + \frac{p_{\mathrm{B}}^2}{2M_{\mathrm{B}}} = \mathrm{KER}$, and therefore the KER velocities assigned to the fragments will be

$$\begin{cases} \mathbf{V}_{\mathrm{A}} = +\mathbf{n}_{AB} \cdot \frac{\sqrt{2\mu \mathrm{KER}}}{M_{\mathrm{A}}} , \\ \mathbf{V}_{\mathrm{B}} = -\mathbf{n}_{\mathrm{AB}} \cdot \frac{\sqrt{2\mu \mathrm{KER}}}{M_{\mathrm{B}}} . \end{cases}$$

2 Computational results

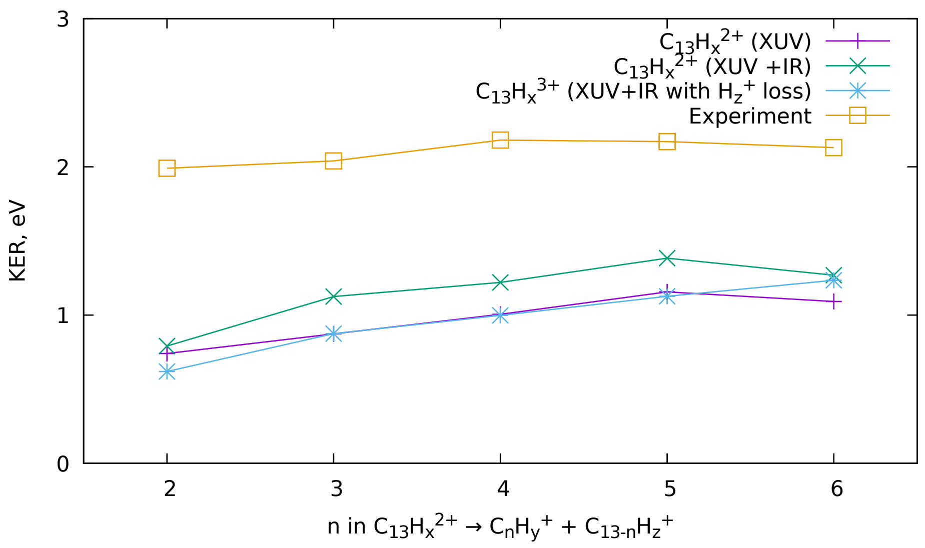

Figure 3: Theoretical and experimental kinetic energy releases (KER) of the (1,1) channels for the fluorene dications. Theoretical values were calculated for the dication produced by the XUV pulse only, for the dication with both the XUV and IR pulses, and for the trication obtained with both XUV and IR following the loss of a H^+ or H_2^+ ion, i.e. a (1,1,1) channel.

We performed four types of simulation of the dissociation dynamics for fluorene ($C_{13}H_{10}$):

- $C_{13}H_{10}{}^{2+}$

  - produced by only the XUV pulse (10927 trajectories),
  - produced by XUV and IR pulses (1835 trajectories);

- ${C_{13}H_{10}}^{3+}$ produced by the XUV and IR pulses (5773 trajectories),
- ${C_{13}T_{10}}^{3+}$ produced by the XUV and IR pulses (13783 trajectories).

The main dissociation dynamics are driven be absorption of an XUV photon, with the IR pulse adding only a small level of additional energy compared to the XUV only simulations. The simulation of the dication with and without IR pulse shows that the kinetic energy release (KER) is only slightly changed with the addition of the IR pulse (Figure 3). The dissociation dynamics of the dication was found to mostly proceed via (1,1) channels with a breakup of the carbon-carbon bonds. The KER in these channels seems to be rather constant at around 1 eV. This is lower than the experimental observation, which is attributed to three main reasons:

- In BOMD, the dissociation occurs only on the ground state potential, whereas in reality the electronically excited states will also produce the (1,1) channel ions, and thus have a higher KER.
- We use the semi-empirical approach to compute the highly energetic conformations of the molecules, which can give some systematic errors in the potential.
- Dissociation potential energy surfaces of the highly-charged cationic states can have a dissociation barrier, which might be missed in simulations due to earlier detection of the dissociation (to avoid the multi-reference character of the bond breaking). Therefore, the results might underestimate KER values.

In case of fluorene interacting with XUV pulse to produce the dissocation from the dication, the distribution and the average kinetic energies (KE) of the individual $C_nH_x^+$ fragments are plotted in Figure 4. It can be seen that the higher mass fragments generally have lower KEs than lower mass fragments, as expected.

In the case of the fluorene trication (${C_{13}H_{10}}^{3+}$), upon interaction with the XUV pulse, the only fragmentations observed within 10 ps were the loss of one or two hydrogen ions. Therefore, to enhance the carbon-carbon fragmentation, the IR pulse was added to the simulation. Carbon backbone fragmentation of the trication mostly progressed through the (1,1,1) channel: first, the ${C_{13}H_{10}}^{3+}$ lost either H^+ or H_2^+, resulting in dicationic ${C_{13}H_9}^{2+}$ and ${C_{13}H_8}^{2+}$, respectively, and the carbon backbone fragmentation continued *via* a (1,1) channel (Figure 3). To quench this process, the protons were replaced by heavier tritium atoms. Of the 13783 ${C_{13}T_{10}}^{3+}$ trajectories, the following three (1,2) channels were observerd:

- $C_{13}H_{10}^{3+} \rightarrow C_7H_6^+ + C_6H_4^{2+}$ (KER = 3.6 eV),
- $C_{13}T_{10}^{3+} \rightarrow C_7T_6^{2+} + C_6T_4^+$ (KER = 3.1 eV),
- $C_{13}T_{10}^{3+} \rightarrow C_{11}T_8^{2+} + C_2T_2^+$ (KER = 2.1 eV).

The first 400 fs of these aBOMD trajectories are also given as movies in the electronic supplementary information. Similar to the (1,1) channels, the (1,2) channels are approximately 1-2 eV lower than the experimental values, which is attributed to the same reasons described above.

References

[1] Stefan Grimme. Towards first principles calculation of electron impact mass spectra of molecules. *Angewandte Chemie International Edition*, 52(24):6306–6312, 2013. doi: https://doi.org/10.1002/anie.201300158. URL https://onlinelibrary.wiley.com/doi/abs/10.1002/anie.201300158.

[2] Christoph Alexander Bauer and Stefan Grimme. How to compute electron ionization mass spectra from first principles. *The Journal of Physical Chemistry A*, 120(21):3755–3766, 2016. doi: 10.1021/acs.jpca.6b02907. URL https://doi.org/10.1021/acs.jpca.6b02907. PMID: 27139033.

[3] Jeroen Koopman and Stefan Grimme. Calculation of electron ionization mass spectra with semiempirical gfnn-xtb methods. *ACS Omega*, 4(12):15120–15133, 2019. doi: 10.1021/acsomega.9b02011. URL https://doi.org/10.1021/acsomega.9b02011. PMID: 31552357.

[4] Denis S. Tikhonov, Amlan Datta, Pragya Chopra, Amanda L. Steber, Bastian Manschwetus, and Melanie Schnell. Approaching black-box calculations of pump-probe fragmentation dynamics of polyatomic molecules. *Zeitschrift für Physikalische Chemie*, 234(7-9):1507–1531, 2020. doi: doi:10.1515/zpch-2020-0009. URL https://doi.org/10.1515/zpch-2020-0009.

[5] Christoph Bannwarth, Sebastian Ehlert, and Stefan Grimme. Gfn2-xtb—an accurate and broadly parametrized self-consistent tight-binding quantum chemical method with multipole electrostatics and density-dependent dispersion contributions. *Journal of Chemical Theory and Computation*, 15(3):1652–1671, 2019. doi: 10.1021/acs.jctc.8b01176. URL https://doi.org/10.1021/acs.jctc.8b01176. PMID: 30741547.

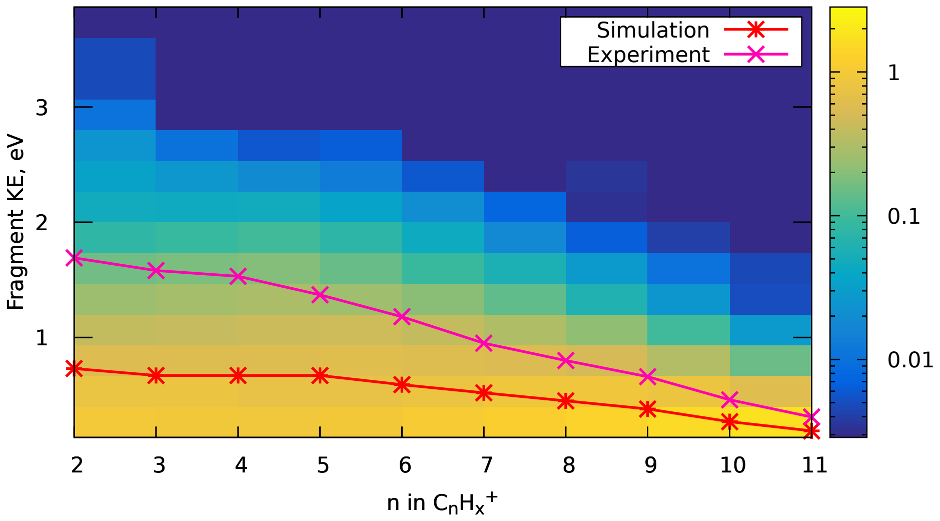

Figure 4: Kinetic energy (KE) distribution map of the fragments $C_nH_x^+$ obtained from the (1,1) fragmentation channels of the fluorene dications resulting from interaction with the XUV pulse. The points in red show the calculated mean KE of the fragments from simulations, points in magenta show the experimentally observed KEs.

[6] Christoph Bannwarth, Eike Caldeweyher, Sebastian Ehlert, Andreas Hansen, Philipp Pracht, Jakob Seibert, Sebastian Spicher, and Stefan Grimme. Extended tight-binding quantum chemistry methods. *WIREs Computational Molecular Science*, 11(2):e1493, 2021. doi: https://doi.org/10.1002/wcms.1493. URL https://wires.onlinelibrary.wiley.com/doi/abs/10.1002/wcms.1493.

[7] Denis S. Tikhonov. Pyramd manual. https://confluence.desy.de/display/CFA/PyRAMD, 2021.

[8] Denis S. Tikhonov. Pyramd. https://stash.desy.de/projects/PYRAMD/repos/pyramd/browse, 2021.

[9] A. S. Cukrowski, S. Fritzsche, and J. Popielawski. Theory of translational energy relaxation in binary mixtures of dilute gases with chemical reaction. *Acta Physica Polonica A*, 82(66):1005–1022, 1992.

[10] M. Hervé, V. Despré, P. Castellanos Nash, V. Loriot, A. Boyer, A. Scognamiglio, G. Karras, R. Brédy, E. Constant, A. G. G. M. Tielens, A. I. Kuleff, and F. Lépine. Ultrafast dynamics of correlation bands following xuv molecular photoionization. *Nature Physics*, 17(3):327–331, Mar 2021. ISSN 1745-2481. doi: 10.1038/s41567-020-01073-3. URL https://doi.org/10.1038/s41567-020-01073-3.

[11] J. W. L. Lee, D. S. Tikhonov, P. Chopra, S. Maclot, A. L. Steber, S. Gruet, F. Allum, R. Boll, X. Cheng, S. Düsterer, B. Erk, D. Garg, L. He, D. Heathcote, M. Johny, M. M. Kazemi, H. Köckert, J. Lahl, A. K. Lemmens, D. Loru, R. Mason, E. Müller, T. Mullins, P. Olshin, C. Passow, J. Peschel, D. Ramm, D. Rompotis, N. Schirmel, S. Trippel, J. Wiese, F. Ziaee, S. Bari, M. Burt, J. Küpper, A. M. Rijs, D. Rolles, S. Techert, P. Eng-Johnsson, M. Brouard, C. Vallance, B. Manschwetus, and M. Schnell. Time-resolved relaxation and fragmentation of polycyclic aromatic hydrocarbons investigated in the ultrafast xuv-ir regime. *Nature Communications*, 12(1):6107, Oct 2021. ISSN 2041-1723. doi: 10.1038/s41467-021-26193-z. URL https://doi.org/10.1038/s41467-021-26193-z.
