# Supplementary material for: The kinetic energy of PAH dication and trication dissociation determined by recoil-frame covariance map imaging
Source: Phys Chem Chem Phys. 2022 Jun 20;24(38):23096–105. doi: 10.1039/d2cp02252d (PMC9533308; doi:10.1039/d2cp02252d)
Supplement: CP-024-D2CP02252D-s002 [file CP-024-D2CP02252D-s002.pdf]

Electronic Supplementary Material (ESI) for Physical Chemistry Chemical Physics.
This journal is © the Owner Societies 2022

- File name: movie_0.mp4
  - $C_{13}H_{10}^{3+} \rightarrow C_7H_6^{+} + C_6H_4^{2+}$ (KER = 3.6 eV)
- File name: movie_1.mp4
  - $C_{13}T_{10}^{3+} \rightarrow C_{11}T_8^{2+} + C_2T_2^{+}$ (KER = 2.1 eV)
- File name: movie_2.mp4
  - $C_{13}T_{10}^{3+} \rightarrow C_7T_6^{2+} + C_6T_4^{+}$ (KER = 3.1 eV)
